# Mapping the landscape of standardized usability questionnaires in healthcare: a 26-year scoping review

**DOI:** 10.3389/fdgth.2026.1938990

**Published:** 2026-08-28

**Authors:** Matthias Fabian Berger, Thomas Augustin, Lars-Peter Kamolz, David Benjamin Lumenta

**Affiliations:** 1 Research Unit for Digital Surgery, Division of Plastic, Aesthetic and Reconstructive Surgery, Department of Surgery, Medical University of Graz, Graz, Austria; 2 Institute for Medical Informatics, Statistics and Documentation, Medical University of Graz, Graz, Austria

**Keywords:** digital health, eHealth, human factors, system usability scale, usability evaluation, user centered design

## Abstract

**Background:**

The evaluation of healthcare technologies utilizing standardized usability questionnaires has grown exponentially, reflecting the increasing importance of user-centered design in eHealth. However, the application of these instruments remains fragmented across clinical specialties and target populations.

**Objective:**

This scoping review synthesizes 26 years of literature to examine how standardized usability questionnaires are applied across distinct use cases, healthcare specialties, and target populations to identify current methodological gaps.

**Methods:**

A search in MEDLINE/PubMed was conducted for articles published between 1999 and June 2025. Studies employing standardized usability questionnaires for evaluating healthcare technologies were included. Data regarding publication year, the specific usability instrument employed, target population, healthcare specialty, and application domain were extracted and analyzed descriptively.

**Results:**

The search yielded 1,342 peer-reviewed articles. The publication volume showed a substantial upward trend, peaking in 2024 with 256 studies. The System Usability Scale (SUS) was the dominant instrument utilized in 92.8 percent of the publications. The Post-Study System Usability Questionnaire (PSSUQ) was the second-most common instrument applied in 6.0 percent of studies. Internal Medicine and Public Health were the most prominent clinical fields. Mobile Apps and Web Tools constituted the most frequently targeted systems, accounting for 41.4 percent. Patients and healthcare professionals represented the primary user groups.

**Conclusions:**

While publication volumes have surged, the field relies heavily on the SUS, limiting the ability to extract diagnostic insights. To ensure that future healthcare technologies are specifically optimized for complex clinical environments, researchers must mature beyond global satisfaction scores and strategically adopt established, comprehensive tools like the PSSUQ.

## 1 Introduction

### 1.1 Usability in healthcare technologies

Usability is defined as the extent to which a system enables specified users to achieve specified goals with effectiveness, efficiency, and satisfaction (1). Usability in healthcare technologies involves more than interface design, as it fundamentally determines how well health IT systems integrate with clinical processes and satisfy users' cognitive needs during daily operations. Ensuring high usability in electronic health records, medical devices, and health apps is essential to prevent inefficiencies, user frustration, burnout and safety hazards (2–5). Ensuring that healthcare technologies are easy to learn, error-tolerant, and satisfactory for clinicians and patients can improve adoption and outcomes (6). However, evaluating usability in healthcare has been challenging due to diverse contexts of use and inconsistent assessment methods. Studies across medical domains have used varied approaches, contributing to a fragmented, discipline-specific evaluation landscape (1). The lack of common metrics limits the comparability and generalizability of health IT usability findings across studies and underscores the need for more standardized and systematic approaches to usability assessment in healthcare (7, 8).

One strategy to bring consistency to usability evaluation is the use of standardized usability questionnaires. These are validated survey instruments with a fixed set of questions and scoring rules designed to quantify users' perceived usability of a system (9). They provide reliable and valid subjective measures of health IT usability and enable consistent evaluation and comparison across studies, as they have undergone extensive psychometric testing including assessments of reliability and validity (10). Standardized questionnaires thus enable objective comparison of usability across different systems or versions, complementing qualitative observations. In usability testing, questionnaires can be administered either at the end of a test session (post-study) or after users complete each task (post-task). Post-study questionnaires capture overall impressions of the system, while post-task questionnaires capture immediate feedback on specific tasks (9). Both types are common in user research and serve different purposes. The advantages of standardized usability questionnaires include improved objectivity, straightforward communication of results, and the availability of normative reference values that facilitate benchmarking across systems and studies (11–13). As self-report measures, these instruments have inherent limitations, as they reflect subjective perceptions rather than providing insight into specific interface issues or their underlying causes (10).

In their seminal work, Sauro and Lewis characterize the following instruments as the most widely adopted standardized questionnaires for post-study and post-task assessment, including their psychometric properties and specific applications in health information technology (HIT) (9).

### 1.2 Post-study questionnaires

Developed by Brooke in 1986, the System Usability Scale (SUS) remains the industry standard for a quick yet remarkably robust global measure of usability. It consists of 10 items rated on a 5-point Likert scale and has consistently demonstrated high reliability (Cronbach's alpha > 0.90) and the ability to distinguish between usable and unusable systems even with small sample sizes (12–14). While a score of 68 is considered the global average (15), healthcare environments target scores above to ensure safety and efficiency for clinical-grade software (16). Although its technology independence and extensive easy benchmarking, the SUS lacks diagnostic insight, identifying a problem existance without specifying its nature or cause (17).

As a more comprehensive alternative, the Post-Study System Usability Questionnaire (PSSUQ), first published by Lewis in 1992 (18), utilizes a 19-item format on a 7-point scale. Unlike the unidimensional SUS, the PSSUQ provides an overall score as well as three subscales that capture System Usefulness (SysUse), Information Quality (InfoQual), and Interface Quality (IntQual), and demonstrates excellent internal consistency, with coefficient alphas between 0.83 and 0.96 for the total scale and subscales (19). In healthcare settings, its separate information-quality and interface-quality subscales are particularly useful for distinguishing usability issues related to data presentation from those related to navigation and interaction (20). However, researchers must remain mindful of potential acquiescence bias, as the entirely positively phrased items may lead respondents to agree reflexively rather than reflect carefully on each statement (21).

Other specialized usability instruments include the Software Usability Measurement Inventory (SUMI) and the Questionnaire for User Interaction Satisfaction (QUIS). SUMI is a standardized 50-item questionnaire designed to assess users' subjective perceptions of software quality across dimensions such as efficiency, affect, helpfulness, control, and learnability (22). Due to its comprehensive structure, SUMI has frequently been applied in human factors and health technology research (23). However, its comparatively long administration time and commercial licensing requirements may limit its practicality in fast-paced clinical usability studies (24).

Similarly, QUIS was developed to evaluate user satisfaction with specific aspects of human–computer interaction, including screen design, terminology, system feedback, learning support, and system capabilities (25). Its modular architecture enables researchers to adapt the questionnaire to different application contexts, such as clinical decision support systems or electronic health records (26). As with the SUS, the QUIS was not explicitly designed to address the broader clinical context (27). Moreover, its considerable length spanning 41 items in the short form and up to 122 in the long form likely encourages researchers in contemporary healthcare settings to favor shorter and more pragmatic usability instruments (28).

### 1.3 Post-task questionnaires

The After Scenario Questionnaire (ASQ) was developed by Lewis in 1991 as a brief 3 item post-task usability instrument for scenario-based usability testing and was later incorporated into the IBM Computer Usability Satisfaction Questionnaires framework. The ASQ comprises three items rated on seven-point Likert scales assessing satisfaction with task completion, time required, and support information. With all coefficient alphas exceeding 0.90, psychometric evaluations demonstrated acceptable reliability, sensitivity, concurrent validity, and a factor structure reflecting scenario-level usability assessment rather than independent item-level constructs (17, 29). ASQ ratings have also been shown to correlate with objective usability metrics such as task completion rates and task times (28). Because the ASQ provides task-specific ratings instead of a single global usability score, it is commonly recommended as a complement to broader post-study questionnaires such as the SUS or the PSSUQ (28, 29).

Usability Magnitude Estimation (UME) applies principles of psychophysical magnitude estimation to usability assessment by asking participants to assign proportional numerical values to perceived task difficulty relative to a reference task, thereby aiming to produce ratio-scale usability data (30, 31). Originally proposed by McGee in 2003 as a potentially more sensitive alternative to ordinal rating scales for detecting relative differences in usability severity (32), the method was subsequently tested in a direct empirical comparison by Sauro and Dumas, who found that participants performed the UME format less easily and that its sensitivity was lower than that of simpler post-task measures (31). Due to its relatively complex administration and non-standard response format, UME has seen limited adoption in applied usability research compared to simpler post-task measures (28).

The Subjective Mental Effort Questionnaire (SMEQ) was developed by Zijlstra and Van Doorn in 1985 as a unidimensional self-report instrument for assessing perceived mental effort during task performance, using a continuous vertical rating scale with anchored descriptors ranging from 0 to 150 (33). Validation studies demonstrated that SMEQ ratings are sensitive to variations in task complexity and mental workload and correlate with other subjective workload measures, supporting its construct validity as an indicator of perceived mental workload (34). Because perceived mental effort is particularly relevant in cognitively demanding clinical environments, mental effort scaling has potential utility in healthcare settings (35). However, unlike widely adopted Likert-based instruments such as the SUS, which benefit from well-established normative benchmarks and extensive psychometric documentation, the SMEQ's non-standard continuous rating format lacks comparable reference values for result interpretation in applied contexts (31, 36).

Introduced by Sauro and Dumas in 2009, the Single Ease Question (SEQ) is a one-item seven-point Likert-type post-task usability measure that demonstrated sensitivity and ease of administration comparable or superior to more complex alternatives such as the SMEQ and UME (31, 37, 38). Despite its brevity, the SEQ has been shown to provide reliable and sensitive assessments of perceived task difficulty and to correlate with objective usability indicators including task completion, task time, and error-related outcomes. Because the SEQ is based on a single item, it does not identify the underlying causes of perceived task difficulty and is therefore commonly used in combination with broader post-session usability instruments such as the SUS or the PSSUQ (28, 31).

Expectation Ratings (ER) were introduced by Albert and Dixon in 2003 as a usability evaluation approach derived from expectancy-disconfirmation theory. Users rate anticipated task difficulty before execution and subsequently compare these pre-task expectations with post-task experience ratings, thereby generating diagnostics on user satisfaction and task performance (39). Experimental research has demonstrated that user expectations can significantly influence subjective usability evaluations, supporting the relevance of expectancy effects in human–computer interaction research (40). However, the additional pre-task rating procedure may influence user expectations and thereby affect subsequent subjective usability evaluations through expectancy or priming effects (40–42). Furthermore, standardized normative benchmarks and interpretation frameworks for expectation–experience discrepancies are still limited, which may contribute to the comparatively infrequent use of expectation-based measures in applied clinical and health information technology usability research (40, 43).

Although robust usability evaluation is critical to the successful implementation of healthcare technologies, the adoption of standardized instruments remains fragmented across clinical specialties and domains of application. While previous reviews have mapped usability evaluation methods in digital health within restricted timelines, a knowledge gap persists regarding the long-term evolution of these practices. Specifically prior research lacked to examine whether the rapid technological transformation of digital health tools has been accompanied by a corresponding evolution in empirical evaluation methodologies. To address this gap, this scoping review synthesizes 26 years of literature to examine how these instruments are selectively applied across distinct use cases and healthcare specialties and target populations. By examining this extended trajectory, our study provides unique insights into how usability testing has changed over time. Furthermore, this review maps longitudinal trends across medical specialties and application domains and primary target populations, thereby providing a highly granular diagnostic map of evaluation preferences and imbalances across the healthcare landscape.

## 2 Methodology

To map usability evaluation practices in healthcare contexts over the past 26 years, we conducted a scoping review.

### 2.1 Information sources and search strategy

Given our focus on usability evaluation within healthcare specialties, we restricted our search to MEDLINE/PubMed. Search terms were derived from the taxonomy of standardized post-study and post-task usability questionnaires as defined by Sauro and Lewis (9), supplemented by the broader terms of eHealth. To ensure a comprehensive capture of the clinical evaluation landscape, the search strategy explicitly incorporated usability, user experience, user satisfaction, and user acceptance as search terms to account for their pragmatic synonymity and interchangeable use in healthcare environments. The search was conducted between April and June 2025 and covered articles published up to June 2025. For MEDLINE/PubMed, the following search string was applied:

((“Usability”[Title/Abstract] OR “User Satisfaction”[Title/Abstract] OR “User Acceptance”[Title/Abstract] OR “User Experience”[Title/Abstract])) AND ((“medicine”[Title/Abstract] OR “medical”[Title/Abstract] OR “health”[Title/Abstract] OR “healthcare”[Title/Abstract] OR “telemedicine”[Title/Abstract] OR “eHealth”[Title/Abstract] OR “mHealth”[Title/Abstract] OR “digital health”[Title/Abstract]) AND (“english”[Language] OR “german”[Language])) AND ((((“Questionnaire for User Interaction Satisfaction”[TIAB] OR QUIS[TIAB]) OR (“Software Usability Measurement Inventory”[TIAB] OR SUMI[TIAB]) OR (“Post-Study System Usability Questionnaire”[TIAB] OR PSSUQ[TIAB]) OR (“System Usability Scale”[TIAB] OR SUS[TIAB]) OR (“After-Scenario Questionnaire”[TIAB] OR ASQ[TIAB]) OR (“Expectation Ratings”[TIAB] OR ER[TIAB]) OR (“Usability Magnitude Estimation”[TIAB] OR UME[TIAB]) OR (“Single Ease Question”[TIAB] OR SEQ[TIAB]) OR (“Subjective Mental Effort Question”[TIAB] OR SMEQ[TIAB]))).

### 2.2 Inclusion and exclusion criteria

Any literature employing standardized usability questionnaires for the evaluation of healthcare technologies across clinical specialties between 1999 and 2025 was considered for inclusion. Studies were included if they met all of the following criteria:

 IC1. The paper reports the use of at least one of the above-mentioned standardized usability questionnaires.
 IC2. The study can be assigned to a defined target population.
 IC3. The study can be assigned to a healthcare specialty.
 IC4. The paper specifies the use case for which the usability questionnaire was administered.
 IC5. The paper is a full or short research article (not solely an abstract or poster presentation).

Studies were excluded if they met any of the following criteria:

 EC1. The publication is a study protocol, systematic review, meta-analysis, commentary, or theoretical paper.
 EC2. The paper is not published in English or German.
 EC3. The record is a duplicate within the dataset.

### 2.3 Search results and selection of literature

**Figure 1:**
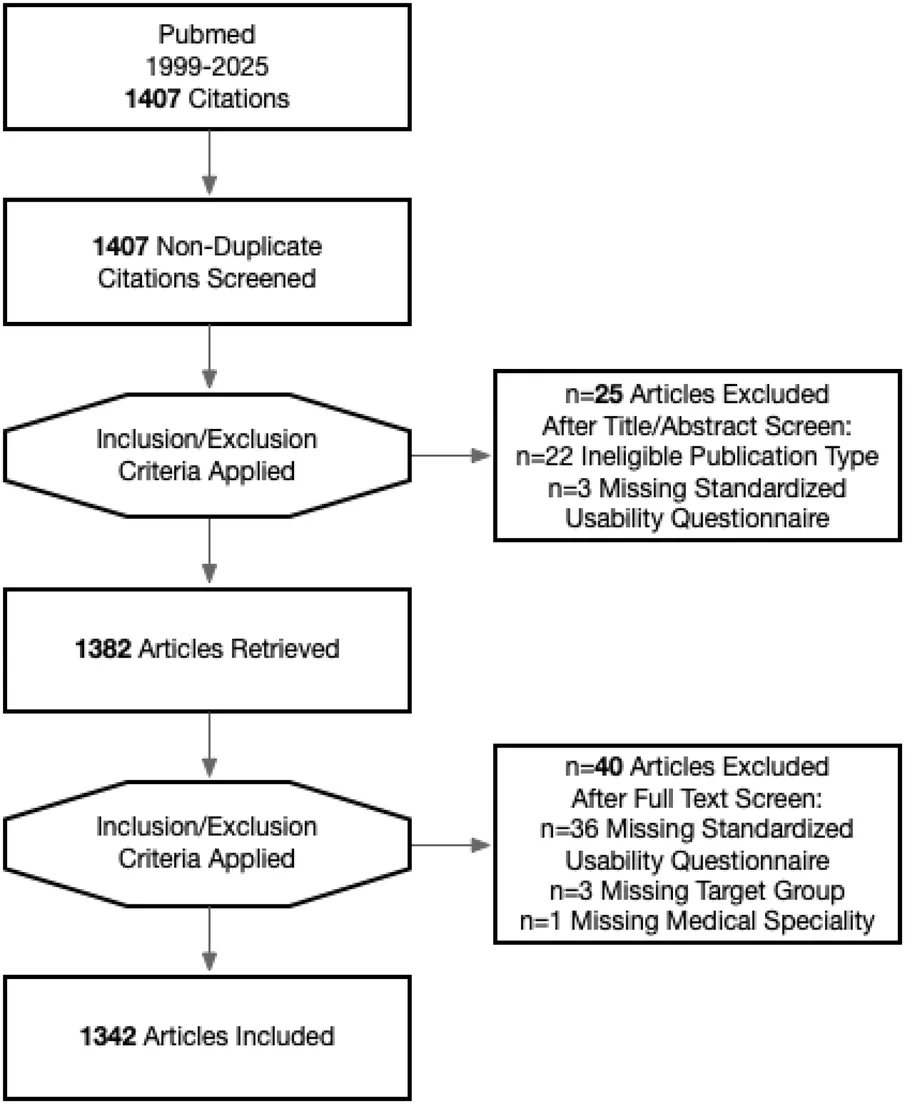
Flowchart of article selection.

A systematic search of MEDLINE/PubMed yielded 1,407 citations for possible inclusion in this scoping review. As no duplicates were identified, all 1,407 citations were screened at the title and abstract level, resulting in the exclusion of 25 articles. The full texts of the remaining 1,382 articles were subsequently assessed against the predefined inclusion and exclusion criteria, leading to the removal of a further 40 papers: 36 were excluded due to a missing usability score, 3 due to an unspecified target group, and 1 due to an unspecified medical specialty. A final sample of 1,342 articles was included in the review. Figure 1 illustrates the systematic process followed for identifying and selecting the literature.

### 2.4 Data extraction and categorisation

From each included article, both general and study-specific data were extracted, encompassing the article title, first author, publication source and year, usability instrument employed, sample size, target population, healthcare specialty, and use case. All extracted data were entered into a structured spreadsheet using Microsoft Excel (Microsoft Corporation, Redmond, Washington, United States of America) to facilitate categorization and comparison of study characteristics, and subsequently exported for statistical analysis using R (R Foundation for Statistical Computing, Vienna, Austria). Given the scoping review design, which prioritizes the mapping of methodological approaches rather than the appraisal of evidence quality, formal critical appraisal of the included articles was not conducted. The detailed classification rules and mapping procedures for healthcare specialties, target populations, and application domains are presented in Supplementary Tables S2–S4.

## 3 Results

### 3.1 Summary of the selected literature

**Figure 2:**
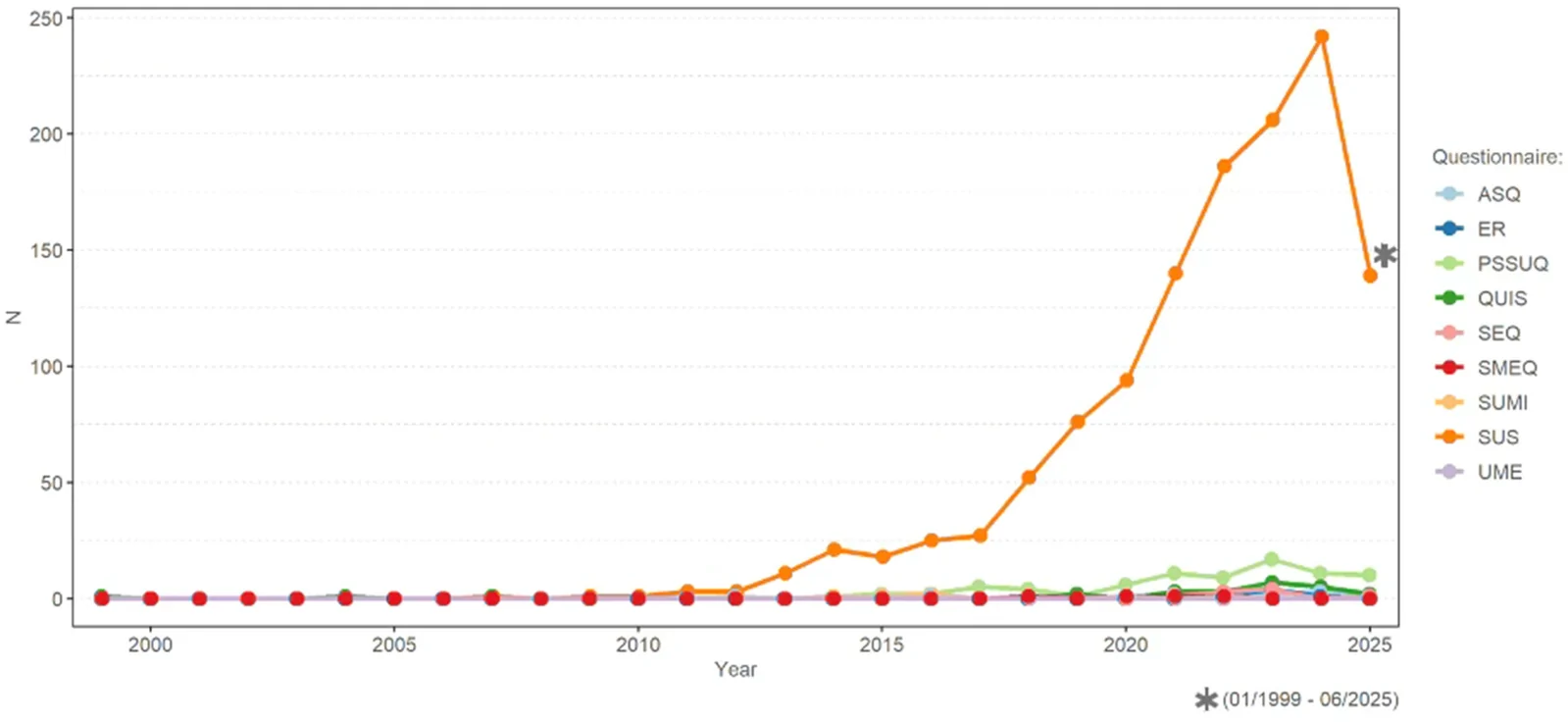
Extracted publications per usability questionnaire per year.

Following the application of the predefined inclusion and exclusion criteria, the systematic search process yielded a comprehensive sample of *n* = 1,342 peer-reviewed articles focusing on the usability evaluation of healthcare technologies. The publication dates of the extracted literature span a 26-year period from 1999 to 2025. A chronological analysis reveals a substantial upward trend in the publication volume over time, reflecting an exponential growth in eHealth research. The vast majority of the studies were published between 2018 and 2024. Specifically, the application of the highly dominant System Usability Scale (SUS) surged from 52 instances in 2018 to a peak of 242 in 2024, accounting for 19.4% of all SUS evaluations (Figure 2; see also Supplementary Table S1 in Supplementary files Section).

### 3.2 Usability testing instruments employed

A variety of standardized quantitative instruments were utilized to evaluate usability across the included studies (Table 1). The System Usability Scale (SUS) was overwhelmingly the most frequently employed questionnaire, utilized in 1,246 of the extracted publications (92.8%). The Post-Study System Usability Questionnaire (PSSUQ) was the second most common instrument, applied in 80 studies (6.0%). Other validated post-study and post-task questionnaires were notably less prevalent in the literature: The Questionnaire for User Interaction Satisfaction (QUIS) was used in 25 studies (1.9%), followed by the After-Scenario Questionnaire (ASQ) with 11 studies (0.8%), and the Single Ease Question (SEQ) with 10 studies (0.8%). More complex or specialized instruments such as the Software Usability Measurement Inventory (SUMI) and Expectation Ratings (ER) were each employed in 6 studies (0.4%), while the Subjective Mental Effort Questionnaire (SMEQ) was utilized in 4 studies (0.3%). Notably, none of the included studies employed Usability Magnitude Estimation (UME) for usability assessment. As several publications employed multiple usability instruments concurrently (e.g., combining the SUS with a post-task questionnaire like the SEQ), the categories are not mutually exclusive. Consequently, the cumulative percentage across all questionnaires exceeds 100%.

**Table 1:** Usability questionnaires employed in the included studies.

Usability Questionnaire	N	%
SUS	1,246	92.8
PSSUQ	80	6.0
QUIS	25	1.9
ASQ	11	0.8
SEQ	10	0.8
SUMI	6	0.4
ER	6	0.4
SMEQ	4	0.3
UME	0	0.0

### 3.3 Healthcare specialties and domains of application

**Figure 3:**
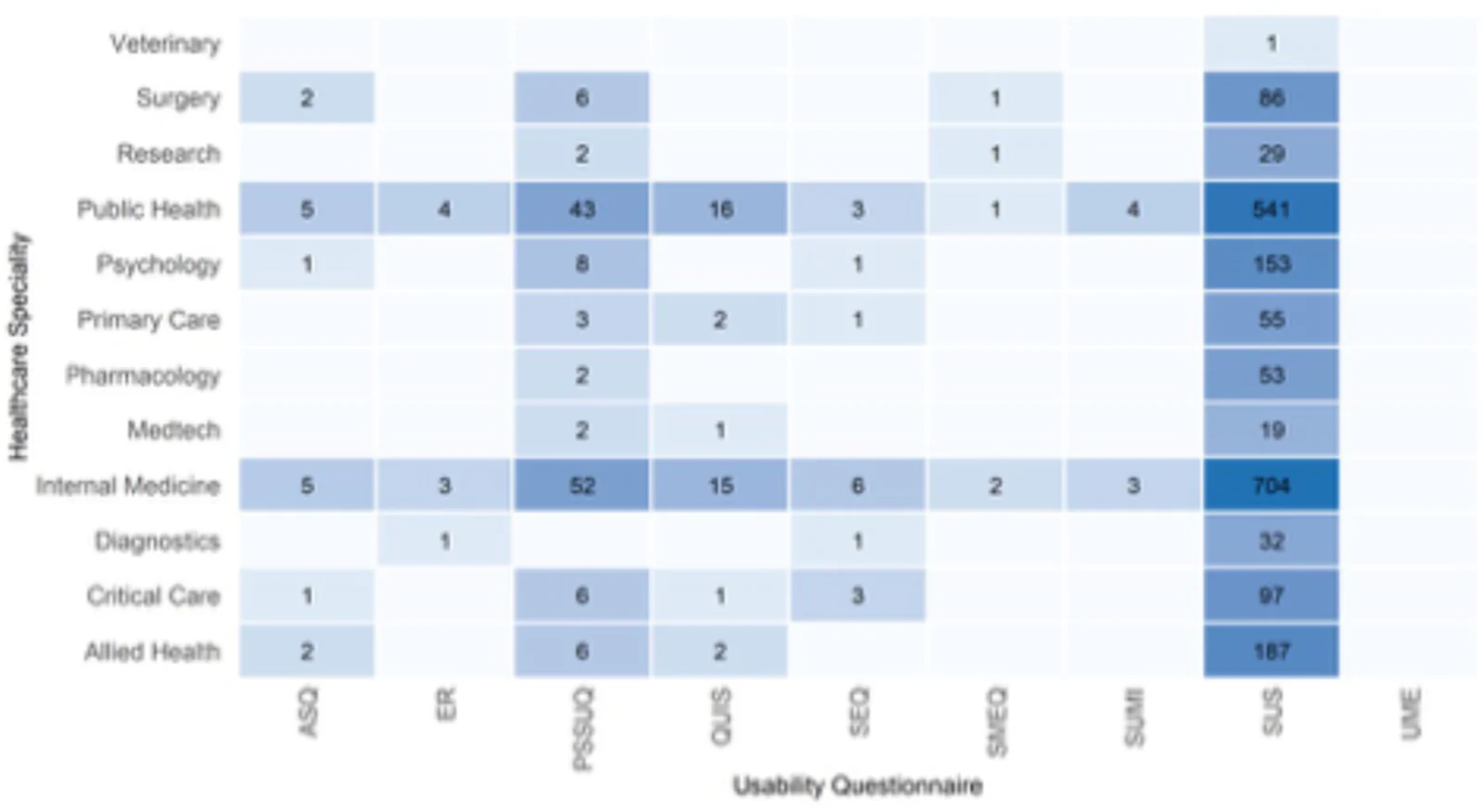
Heatmap healthcare specialties vs. Usability Questionnaires.

The application of standardized usability questionnaires was mapped across various defined healthcare specialties (Table 2). Because contemporary digital health research frequently involves interdisciplinary approaches, studies encompassing multiple clinical fields were assigned to all applicable healthcare specialties using a multilabel classification approach. Consequently, the cumulative percentage across all medical disciplines exceeds 100 percent. Internal Medicine represented the most prominent clinical field, encompassing 766 studies (57.1%). This was followed by Public Health (*n* = 592, 44.1%), Allied Health (*n* = 195, 14.5%), and Psychology (*n* = 161, 12.0%). Other significant disciplines conducting formalized usability evaluations included Critical Care (*n* = 102, 7.6%), Surgery (*n* = 91, 6.8%), Primary Care (*n* = 59, 4.4%), and Pharmacology (*n* = 55, 4.1%). Fields such as Diagnostics (*n* = 32, 2.4%), Research (*n* = 30, 2.2%), Medical Technology (*n* = 20, 1.5%), and Veterinary Medicine (*n* = 1, 0.1%) constituted a smaller proportion of the literature. Cross-referencing the employed usability instruments with the respective healthcare specialties (Figure 3), the System Usability Scale (SUS) demonstrated dominance across all medical fields. For instance, within Internal Medicine, the SUS was utilized in 704 instances, far exceeding the PSSUQ, which was applied only 52 times. Similarly, in Public Health, the SUS accounted for 541 applications.

**Table 2:** Evaluated healthcare specialities.

Healthcare Speciality	N	%
Internal Medicine	766	57.1
Public Health	592	44.1
Allied Health	195	14.5
Psychology	161	12.0
Critical Care	102	7.6
Surgery	91	6.8
Primary Care	59	4.4
Pharmacology	55	4.1
Diagnostics	32	2.4
Research	30	2.2
Medtech	20	1.5
Veterinary	1	0.1

Regarding the specific technological applications evaluated (Table 3), Mobile Apps & Web Tools were the most frequently targeted systems, accounting for 556 studies (41.4%). Patient Care & Telehealth applications (*n* = 399, 29.7%) and technologies developed for Research, Education & Public Health (*n* = 349, 26.0%) also formed a substantial portion of the evaluated use cases. Furthermore, usability testing was regularly applied to Rehabilitation & Training systems (*n* = 292, 21.8%), Decision Support & Safety tools (*n* = 161, 12.0%), and Clinical Systems & Clinical Workflows (*n* = 125, 9.3%). XR & Simulation and Surgical Tools each represented approximately 7.9% to 8.1% of the evaluated applications, followed by Devices & Wearables (*n* = 79, 5.9%) and Diagnostics (*n* = 70, 5.2%). Due to the multifaceted nature of digital health interventions, single publications frequently evaluated technologies that addressed multiple application domains simultaneously. Therefore, the sum of percentages across all domains exceeds 100%.

**Table 3:** Domains of application.

Application Domain	N	%
Mobile Apps & Web Tools	556	41.4
Patient Care & Telehealth	399	29.7
Research, Education & Public Health	349	26.0
Rehabilitation & Training	292	21.8
Decision Support & Safety	161	12.0
Clinical Systems & Clinical Workflow	125	9.3
XR & Simulation	108	8.1
Surgical Tools	106	7.9
Devices & Wearables	79	5.9
Diagnostics	70	5.2

**Figure 4:**
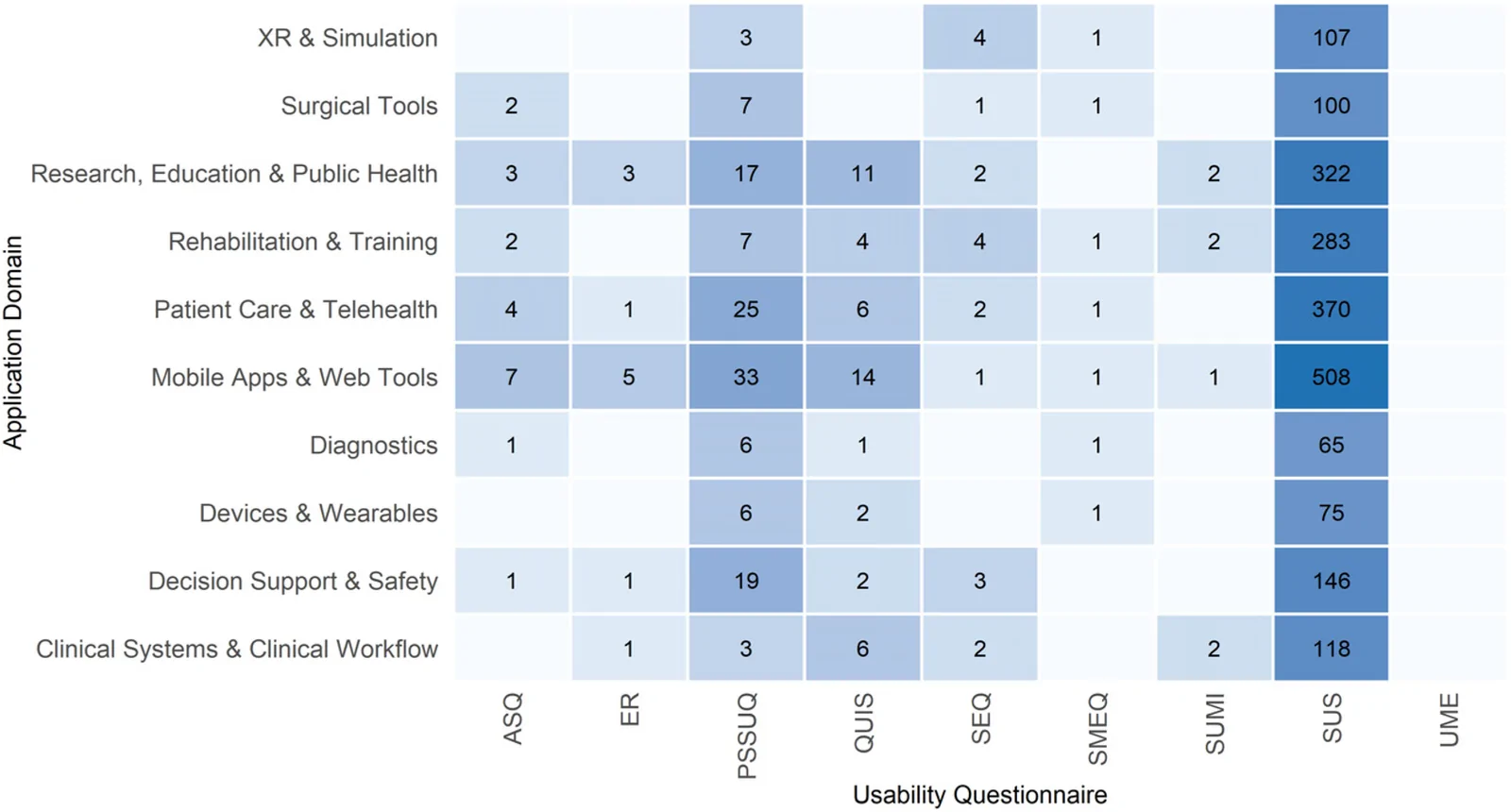
Heatmap application domains vs. Usability Questionnaires.

**Figure 5:**
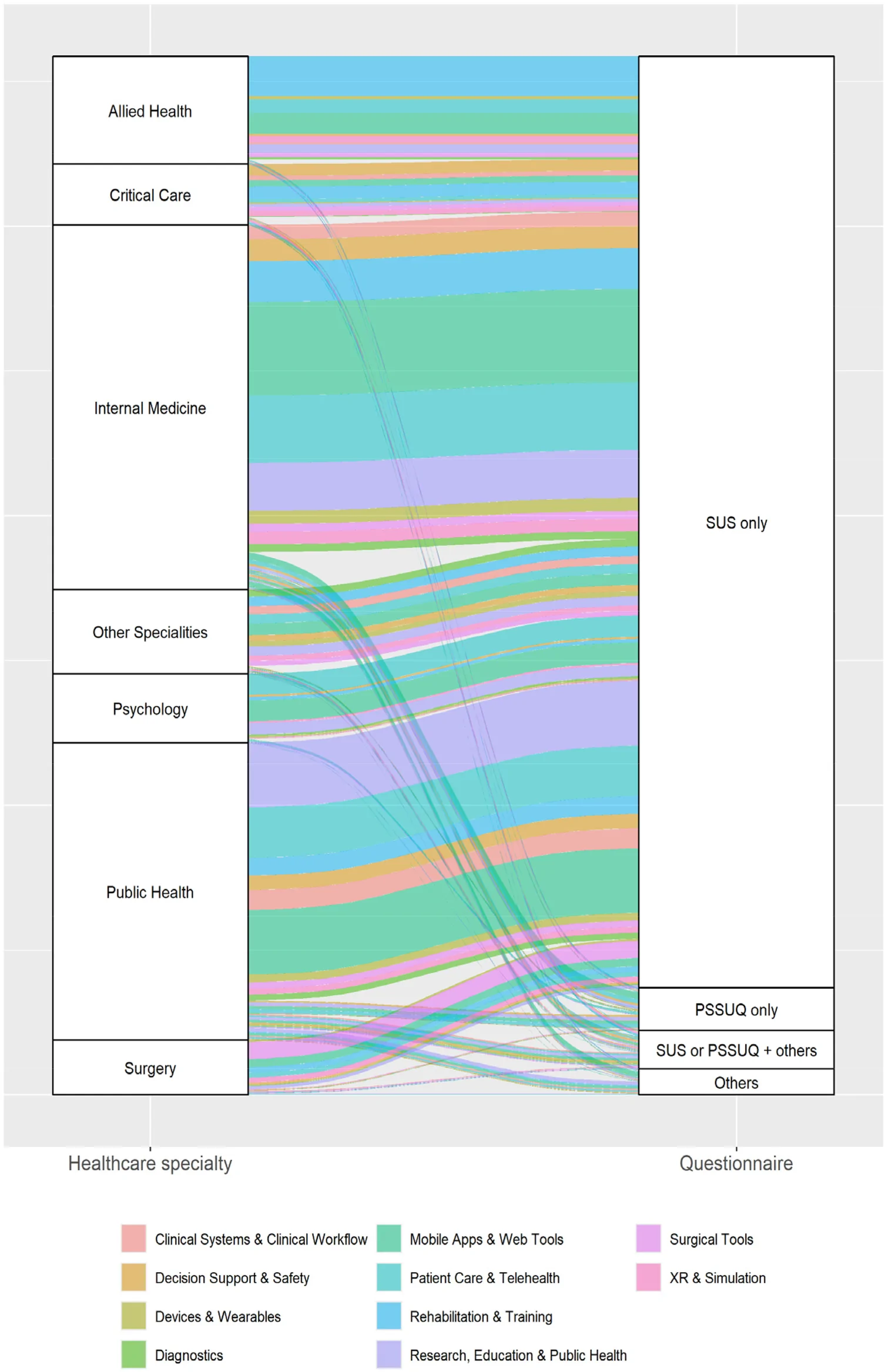
Sankey diagram illustrating the flow from healthcare specialties to usability questionnaires, color-coded by application domain.

Further analysis of the application domains via cross-tabulation (Figure 4) underscores the predominant use of the SUS across most digital health interventions. For example, it was applied in 508 out of 556 studies evaluating Mobile Apps & Web Tools. However, a more detailed examination reveals distinct methodological choices within complex clinical systems. Notably, in the evaluation of Decision Support & Safety tools, the Post-Study System Usability Questionnaire (PSSUQ) was utilized in 11.8% of the studies (*n* = 19). This represents a substantially higher adoption rate compared to its overall average of 6.0%. The distribution and flow from healthcare specialties to specific questionnaire categories, stratified by their respective application domains, are visualized in the Sankey diagram (Figure 5).

### 3.4 Target populations in usability testing

The analysis identified distinct target populations participating in the usability evaluation processes (Table 4). Patients constituted the primary user group, being involved as evaluation participants in 736 studies (54.8%). Healthcare Professionals (HCPs) represented the second largest demographic, participating in 581 of the usability assessments (43.3%). Furthermore, the general public was involved in 183 studies (13.6%), whereas academic personnel (*n* = 141, 10.5%), IT specialists (*n* = 92, 6.9%), and management staff (*n* = 20, 1.5%) were engaged less frequently. Since a considerable subset of studies utilized multi-stakeholder approaches by evaluating usability with more than one target group simultaneously (e.g., engaging both patients and healthcare professionals), these categories are not mutually exclusive, leading to a cumulative total exceeding 100%.

**Table 4:** Target populations in usability testing.

Target Group	N	%
Patients	736	54.8
Healthcare Professionals	581	43.3
Public	183	13.6
Academic	141	10.5
IT	92	6.9
Management	20	1.5

**Figure 6:**
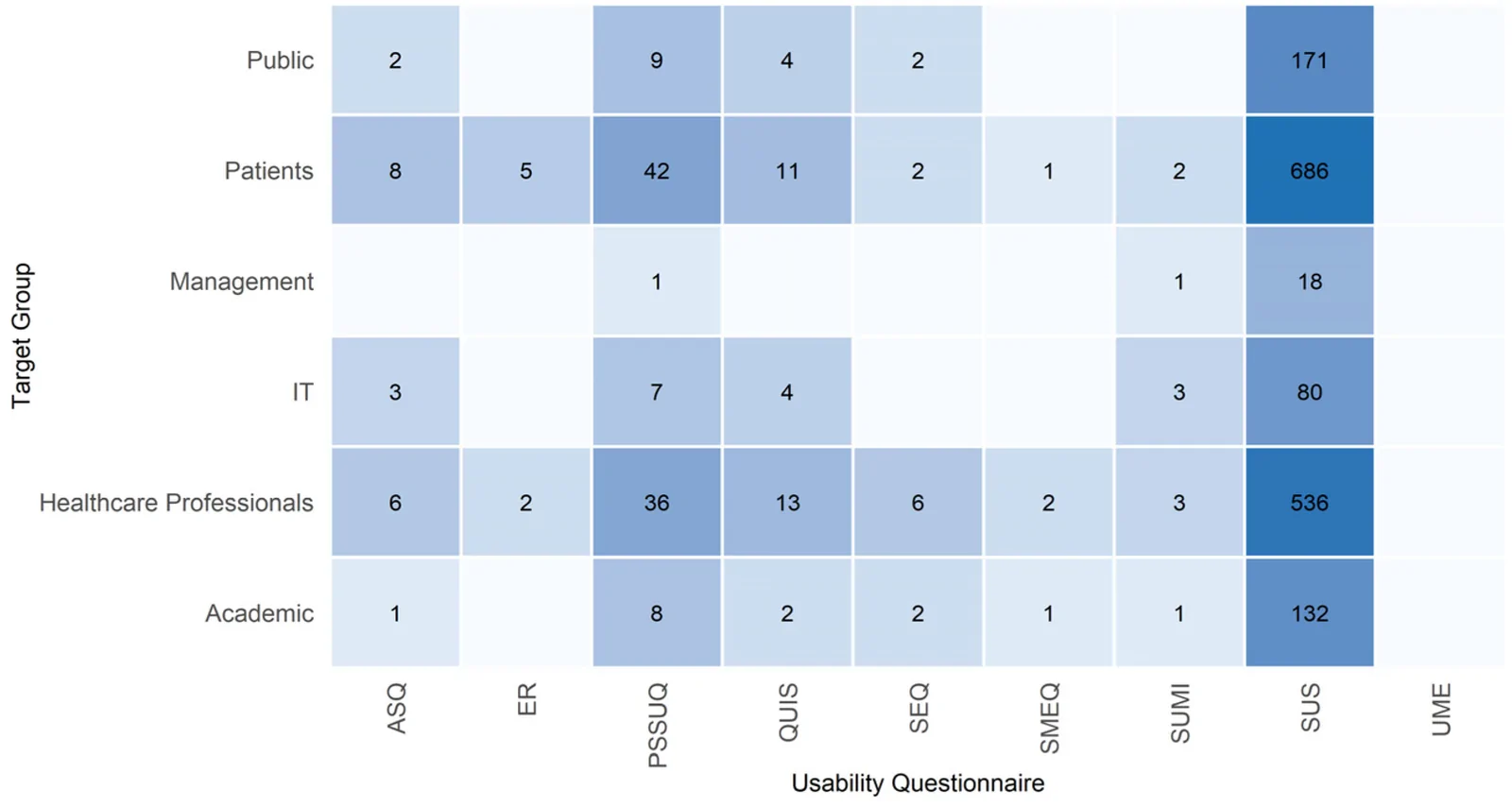
Heatmap target groups vs. Usability Questionnaires.

Further cross-tabulation of the usability questionnaires against the targeted user populations (Figure 6) revealed that the SUS was the preferred instrument regardless of the stakeholder group. It was administered to patients in 686 instances and to healthcare professionals in 536 instances. Interestingly, while patient evaluations relied almost exclusively on the SUS, usability assessments involving technical personnel demonstrated a slightly more diversified methodological approach. When IT specialists were the target population, the application of diagnostic instruments such as the PSSUQ (7.6%, *n* = 7), QUIS (4.3%, *n* = 4), and SUMI (3.3%, *n* = 3) saw a relative proportional increase compared to patient-facing evaluations. This indicates a subtle shift towards more comprehensive usability metrics when systems are evaluated by users with high technical expertise.

## 4 Discussion

### 4.1 Key findings

This scoping review mapped the landscape of standardized usability evaluation in healthcare technologies over a 26-year period, identifying an exponential growth in published research. The surging number of publications accounting the SUS, which peaked in 2024 with 242 studies, mirrors the rapid integration of eHealth solutions into clinical practice and patient self-management. A central finding of this review is the dominance of the SUS, which was employed in 92.8% of the 1,342 included studies. This confirms its status as the industry standard for a quick and robust global measure of usability (13). This longitudinal trajectory highlights a key novelty of our study, demonstrating that the popularity of the SUS is not a temporary trend but a sustained preference that has intensified over nearly three decades. Rather than indicating a mere methodological limitation, this enduring reliance underscores the psychometric robustness and versatility of the instrument as clinical technologies transitioned from simple web portals to complex mobile platforms. In contrast to this global measure, more diagnostic or task specific instruments such as the PSSUQ or the QUIS were utilized far less frequently, appearing in only 6.0% and 1.9% of the studies, respectively. While the SUS provides a reliable global benchmark and benefits from extensive normative data, it lacks the diagnostic insight required to identify the specific nature or underlying causes of usability problems (17). Interestingly, our dataset reveals distinct methodological trends across healthcare specialties, application domains, and target populations. Standardized usability evaluation remains highly concentrated in clinical specialties like Internal Medicine, which accounted for 57.1% of the studies, and Public Health at 44.1%. Regarding application domains, while Mobile Apps and Web Tools dominated at 41.4%, high risk technological applications like Surgical Tools (7.9%) and Diagnostics (5.2%) are underrepresented. Similarly, target group trends show a clear discrepancy, as evaluations involving Patients at 54.8% and Healthcare Professionals at 43.3% rely almost exclusively on the SUS, while assessments involving highly technical personnel like IT specialists exhibit a more diversified approach through the adoption of multi-dimensional diagnostic instruments like the PSSUQ.

### 4.2 Methodological gaps and the evolution of usability instruments

Despite the critical importance of evaluating complex healthcare workflows, the application of multifaceted usability instruments remains disproportionately low. The review revealed insights where researchers attempted to address the limitations of the SUS through various modifications and adaptations rather than employing more comprehensive questionnaires. For instance, the Intervention Usability Scale (IUS) replaced the term system with intervention to better suit clinical contexts while maintaining the original structure of the instrument (44). Other adaptations focused on linguistic accessibility such as the Skala Kebolehgunaan Aplikasi Mudah Alih (SKAMA) instrument which provided a validated Malay translation (45). To reduce respondent burden, several studies employed abbreviated versions like the Usability Metric for User Experience (UMUX) which rely on a corrective regression formula to estimate the overall usability score (46, 47). Beyond minimizing respondent burden, the four-item UMUX and its two-item derivative, the UMUX-LITE, reflect a contemporary shift from pure usability toward broader user experience evaluation. By focusing on perceived usefulness and ease of use, they provide a highly robust, short surrogate for the SUS (48).

Similarly, simplified versions were introduced specifically for older adults and individuals with cognitive impairments to ensure better comprehension (49). Furthermore, Marcelo et al. and Avila et al. utilized truncated versions of the original SUS by analyzing only eight out of the ten items and calculating modified percentage scores (50, 51). While these modifications demonstrate a clear need for flexible usability assessment tools, they compromise the standardized comparability of the original instrument.

In contrast, minor lexical adaptations represent a less intrusive approach, as the SUS remains robust to small wording changes without affecting its validity or psychometric integrity (15, 36). The adaptation of generic terms such as “system” to domain-specific terminology (e.g., “electronic health record” or “mobile application”) is a common practice to reduce ambiguity in clinical contexts. Adding supplementary explanatory prompts for complex clinical tasks provides essential context for healthcare professionals and patients alike. Synthesizing these adaptations provides actionable pathways to tailor specialized medical workflows while preserving standardized comparability.

Future research should therefore advocate for a more balanced or combined methodological approach, because because healthcare usability barriers often arise from complex clinical workflow mismatches, excessive cognitive workload, and latent safety risks. Task specific instruments such as the ASQ and the SEQ are highly sensitive and correlate with objective usability metrics, yet they were scarcely adopted. Highly complex methods like the UME saw zero adoption in the evaluated dataset. This is likely due to their non-standard response formats and difficult administration in fast paced clinical settings. Combining global metrics with specific task level questionnaires or mixed methods approaches incorporating qualitative assessments such as Think-Aloud protocols could yield deeper and more actionable insights for technology developers (52).

The rapid expansion of artificial intelligence in medicine demands a fundamental shift in how usability is conceptualized and evaluated. Because traditional usability instruments fail to capture the dynamic and adaptive nature of machine learning technologies (53), future evaluation strategies should align with international consensus guidelines such as the FUTURE AI framework. This framework emphasizes five core evaluation dimensions comprising clinical workflow integration, human AI interaction and oversight, user training, real world user experience, and empirical clinical utility and safety. Within these dimensions, addressing algorithmic opacity through system explainability is crucial to ensure that model outputs remain clinically meaningful without inducing automation bias or cognitive overreliance among healthcare providers (54).

### 4.3 Implications for healthcare technology development

The findings bear significant implications for the design and implementation of digital health technologies. Usability is not merely an aesthetic concern but fundamentally impacts patient safety, workflow efficiency, and clinician well-being. Poor usability in Electronic Health Records (EHRs) and clinical decision support systems is a recognized driver of professional burnout among healthcare providers (55). The fact that Internal Medicine (57.1%) and Public Health (44.1%) heavily utilized standardized questionnaires is encouraging, as it demonstrates a growing awareness of usability in these disciplines. However, surgical disciplines (6.8%), diagnostic fields (2.4%) and medical technology (1.5%) represented only a fraction of the usability literature. Given the high-risk nature of these specialties, stakeholders and policymakers must incentivize rigorous, standardized usability testing in these underrepresented areas to prevent safety hazards and ensure optimal clinical integration.

Our results align with Maramba et al. as they analyzed usability evaluation methods in eHealth between 2014 and 2017 and similarly identified questionnaires particularly the SUS as the most prevalent evaluation method. While such global questionnaires provide an overall measure of usability they often fail to pinpoint the exact interface problems that require modification (56). Our data covering 26 years reveals that this methodological reliance on the SUS has only intensified over time rather than diversifying into more diagnostic instruments. This indicates a persistent methodological stagnation where rapid global metrics overshadow detailed diagnostic approaches.

Furthermore, our findings highlight a discrepancy between the exponential growth of available commercial health applications and the comparatively slow increase in published peer reviewed usability evaluations. Although usability evaluations are widely conducted in industry, their methods and findings are rarely reported in the literature. This gap may reflect concerns about intellectual property and competitive advantage, which limits knowledge transfer between industry and academia. Consequently, the scientific community remains largely unaware of the usability standards applied to thousands of health applications currently available. To bridge this gap and facilitate successful clinical integration developers must look toward established regulatory guidelines. The Evidence Standards Framework for Digital Health Technologies published by the National Institute for Health and Care Excellence (NICE) demands published evidence of user involvement and user satisfaction for clinical adoption (56, 57).

To optimize evaluation efficiency, a risk stratified instrument matching strategy should be adopted. For low-risk digital health technologies, such as patient education applications or wellness trackers, a rapid global metric like the SUS or short surrogates (e.g., UMUX) are sufficient to ensure basic acceptability. For moderate risk systems, including remote patient monitoring and telemedicine platforms, global metrics should be supplemented with task specific measures like the SEQ or ASQ to identify localized operational bottlenecks. For high-risk clinical technologies, such as clinical decision support systems and robotic surgical tools, a multi method framework utilizing the diagnostic PSSUQ alongside objective clinical workflow simulation and safety tests is mandatory. This combined approach would ensure that systems are not just broadly acceptable but optimized to reduce cognitive load and prevent critical errors in high stakes clinical environments (58, 59).

### 4.4 Limitations

The literature search was restricted to MEDLINE and PubMed to specifically target usability studies representative of medical and clinical research. Consequently, relevant usability studies published exclusively in engineering or computer science and human computer interaction journals might have been omitted. Second the search was limited to publications in English and German which represents a geographic bias, potentially omitting relevant studies from regions such as Asia and South America. Since the SUS has been officially translated and validated in over twenty languages, our language restriction likely led to the exclusion of important cultural and linguistic adaptations of usability assessment practices. The publication drop observed in 2025 is an artifact of the data collection timeline as the search was conducted in mid 2025 and does not reflect a decline in research output. Finally, studies employing heavily modified or unvalidated versions of the predefined questionnaires were excluded meaning that informal usability testing practices were not entirely captured in the quantitative synthesis of this review.

## 5 Conclusions

The evaluation of healthcare technologies utilizing standardized usability questionnaires has grown exponentially, reflecting the increasing importance of user centered design in eHealth. The System Usability Scale remains the primary instrument across virtually all clinical specialties and target populations. However, researchers frequently resort to structural modifications of this scale to accommodate specific user needs or time constraints, which consequently limits standardized comparability across studies. Looking forward, the clinical context does not require the invention of new usability questionnaires or a further fragmentation of existing scales. Instead, the field must strategically adopt established and comprehensive tools. Specifically, the Post Study System Usability Questionnaire must be highlighted as an instrument to evaluate complex clinical systems because it provides deep diagnostic insights into interface quality and information structure. Furthermore, it is crucial to maintain a clear methodological boundary between usability evaluation and behavioral science. While behavioral science evaluates personal habits, usability testing measures the efficiency, effectiveness, and satisfaction of the human computer interaction. Embracing established diagnostic instruments while strictly maintaining this focus on interface interaction will be crucial for developing safe, efficient, and user friendly digital health interventions that genuinely support clinical workflows and patient care.
