# Supplementary material for: Mapping the landscape of standardized usability questionnaires in healthcare: a 26-year scoping review
Source: Front Digit Health. 2026 Aug 28;8:1938990. doi: 10.3389/fdgth.2026.1938990 (PMC13562835; doi:10.3389/fdgth.2026.1938990)
Supplement: Supplementary file 1 [file Datasheet1.pdf]

Supplementary Files

Table 1: Extracted Publications per Usability Questionnaires per Year

Usability Questionnaire	Year	N	Total	%
SUS	2007	1	1,246	0.1
SUS	2009	1	1,246	0.1
SUS	2010	1	1,246	0.1
SUS	2011	3	1,246	0.2
SUS	2012	3	1,246	0.2
SUS	2013	11	1,246	0.9
SUS	2014	21	1,246	1.7
SUS	2015	18	1,246	1.4
SUS	2016	25	1,246	2.0
SUS	2017	27	1,246	2.2
SUS	2018	52	1,246	4.2
SUS	2019	76	1,246	6.1
SUS	2020	94	1,246	7.5
SUS	2021	140	1,246	11.2
SUS	2022	186	1,246	14.9
SUS	2023	206	1,246	16.5
SUS	2024	242	1,246	19.4
SUS	2025	139	1,246	11.2
PSSUQ	2012	1	80	1.2
PSSUQ	2014	1	80	1.2
PSSUQ	2015	2	80	2.5
PSSUQ	2016	2	80	2.5
PSSUQ	2017	5	80	6.2
PSSUQ	2018	4	80	5.0
PSSUQ	2019	1	80	1.2
PSSUQ	2020	6	80	7.5
PSSUQ	2021	11	80	13.8
PSSUQ	2022	9	80	11.2
PSSUQ	2023	17	80	21.2
PSSUQ	2024	11	80	13.8
PSSUQ	2025	10	80	12.5
QUIS	1999	1	25	4.0
QUIS	2004	1	25	4.0
QUIS	2007	1	25	4.0
QUIS	2019	2	25	8.0
QUIS	2021	3	25	12.0
QUIS	2022	3	25	12.0
QUIS	2023	7	25	28.0
QUIS	2024	5	25	20.0
QUIS	2025	2	25	8.0
SUMI	2014	1	6	16.7
SUMI	2015	1	6	16.7
SUMI	2016	2	6	33.3
SUMI	2020	1	6	16.7
SUMI	2022	1	6	16.7
SUMI	2025	0	6	0.0
ASQ	2012	1	11	9.1
ASQ	2016	1	11	9.1
ASQ	2020	1	11	9.1
ASQ	2021	2	11	18.2
ASQ	2023	2	11	18.2
ASQ	2024	3	11	27.3
ASQ	2025	1	11	9.1
ER	2022	1	6	16.7
ER	2023	4	6	66.7
ER	2024	1	6	16.7
ER	2025	0	6	0.0
UME	2025	0	0	
SEQ	2018	1	10	10.0
SEQ	2021	1	10	10.0
SEQ	2022	3	10	30.0

Usability Questionnaire	Year	N	Total	%
SEQ	2023	4	10	40.0
SEQ	2025	1	10	10.0
SMEQ	2018	1	4	25.0
SMEQ	2020	1	4	25.0
SMEQ	2021	1	4	25.0
SMEQ	2022	1	4	25.0
SMEQ	2025	0	4	0.0

Table 2: Mapping of Clinical Specialties to Primary Healthcare Domains

Primary Domain	Included Specialties
PrimaryCare	Primary Care
Surgery	Surgery; Vascular Surgery; Cardiac Surgery; Oral and Maxillofacial Surgery; Neurosurgery; Orthopedics; Plastic Surgery; Thoracic Surgery; Trauma Surgery; Transplant Medicine
Medicine	Internal Medicine; Gastroenterology; Cardiology; Pulmonology; Nephrology; Endocrinology; Hematology; Oncology; Rheumatology; Immunology; Infectious Diseases; Neonatology; Pediatrics; Geriatrics; Gynecology; Hepatology; Dermatology; Otorhinolaryngology; Ophthalmology; Urology; Sexual Medicine; Pain Medicine; Sleep Medicine; Palliative Medicine; Neurology; Nutritional Medicine; Vascular Medicine; Dentistry; Sports Medicine
Emergency	Anesthesiology; Intensive Care Medicine; Emergency Medicine
Psychology	Psychiatry; Psychology
AlliedHealth	Nursing; Occupational Therapy; Physical Therapy; Speech Therapy; Rehabilitation; Social Work
Diagnostics	Laboratory Medicine; Microbiology; Nuclear Medicine; Human Genetics; Radiology; Forensic Medicine
Pharmacology	Pharmacology
PublicHealth	Public Health; Occupational Medicine; Military Medicine; Health Informatics; Digital Health
Research	Anatomy; Research; Ethics; Medical Education
MedTech	Medical Technology
Veterinary	Veterinary Medicine

Table 3: Classification of Targeted User Groups to Primary Target Populations

Primary Domain	Included Application Types
Clinical Systems & Clinical Workflow	Electronic Medical Record; Clinical Workflow Management System; Clinical Terminology System; Clinical Visualization Dashboard; Health Information Exchange Platform; Emergency Department Management System; Computerized Provider Order Entry
Decision Support & Safety	Clinical Guideline Interface; Clinical Decision Support System; Patient Safety Assessment Tool
Patient Care & Telehealth	Patient Self-Management Application; Patient Support Application; Caregiver Support Application; Telemedicine Consultation Platform; Digital Symptom Tracking System; Patient Health Portal
Rehabilitation & Training	Digital Rehabilitation Platform; Movement Tracking System; Therapeutic Exercise Game; Cognitive Training Application
XR & Simulation	Virtual Reality Environment; Three-Dimensional Visualization Model; Augmented Reality Interface; Medical Simulation Training System; Mixed Reality Interface
Mobile Apps & Web Tools	Health Management Platform; Mobile Health Application; Web-Based Health Platform; Conversational Artificial Intelligence Agent
Devices & Wearables	Medical Sensor Device; Automated Infusion Pump; Wearable Continuous Monitor; Robotic Exoskeleton; Portable Medical Device
Diagnostics	Digital Screening Tool; Point of Care Testing Device; Medical Imaging Analysis Software; Laboratory Information System
Surgical Tools	Robotic Surgical Interface; Surgical Planning Interface
Research, Education & Public Health	Digital Medical Education Platform; Research Data Repository; Public Health Surveillance System

Table 4: Categorization of Digital Health Technologies to Primary Application Domains

Primary Domain	Included Populations
Healthcare Professionals	Physicians; Nursing Staff; Pharmacists; Pharmaceutical Scientists; Occupational Therapists; Physical Therapists; Psychologists; Therapists; Emergency Medical Services
Patients & Public	Patients; Healthy Participants; Relatives
IT & Technical Staff	IT Experts; Technicians
Academic & Research Staff	Researchers; Students; Educators
Management & Administration	Management
